# Association of Multiple Sclerosis Phenotypes with Single Nucleotide Polymorphisms of *IL7R*, *LAG3*, and *CD40* Genes in a Jordanian Population: A Genotype-Phenotype Study

**DOI:** 10.3390/biom10030356

**Published:** 2020-02-26

**Authors:** Laith AL-Eitan, Malak Al Qudah, Majdi Al Qawasmeh

**Affiliations:** 1 Department of Applied Biological Sciences, Jordan University of Science and Technology, Irbid 22110, Jordan; msalqudah15@sci.just.edu.jo; 2 Department of Biotechnology and Genetic Engineering, Jordan University of Science and Technology, Irbid 22110, Jordan; 3 Department of Neuroscience, Division of Neurology, Faculty of Medicine, Jordan University of Science and Technology, Irbid 22110, Jordan; m0alqawasmeh@just.edu.jo

**Keywords:** multiple sclerosis, phenotype, *IL7R*, *LAG3*, *CD40*, Jordan

## Abstract

It is thought that genetic variations play a vital role in the Multiple Sclerosis (MS) etiology. However, the role of genetic factors that influence the clinical features of MS remains unclear. We investigated the correlation between 21 single nucleotide polymorphisms within three genes (*IL7R*, *LAG3*, and *CD40*) and MS clinical characteristics in the Jordanian population. Blood samples and clinical phenotypic data were collected from 218 Arab Jordanian MS patients, vitamin D was measured, genomic DNA was extracted, and genotyping of the candidate genes’ polymorphisms were analyzed using the Sequenom MassARRAY^®^ system. The association of these single nucleotide polymorphisms (SNPs) with MS was performed using a Chi-square, Fisher exact test, and one-way ANOVA. We found a significant association between vitamin D deficiency and three SNPs of the *IL7R* gene, namely rs987107 (*P*-value = 0.047), rs3194051 (*P*-value = 0.03), and rs1494571 (*P*-value = 0.036), in addition to two SNPs of *CD40*, namely rs1883832 and rs6074022 (*P*-value = 0.049 for both). rs3194051 of the *IL7R* gene (*P*-value = 0.003) and rs1922452 of the *LAG3* gene (*P*-value = 0.028) were strongly associated with comorbidity. The number of relapses before drug onset was found to be correlated with IL7R SNPs rs969128 (*P*-value = 0.04) and rs1494555 (*P*-value = 0.027), whereas the expanded disability status scale (EDSS) was associated with rs1494555 polymorphism of *IL7R* gene (*P*-value = 0.026). Current findings indicate important correlations between certain SNPs and the risk of various phenotypes of multiple sclerosis in the Jordanian community. Therefore, this will not only contribute to the understanding of MS, but will also assist with the development of personalized treatment procedures.

## 1. Introduction

Multiple sclerosis (MS) is a chronic inflammatory autoimmune disorder that is characterized by immune cells infiltrating by self-reactive T cell-mediated damage in the central nervous system (CNS) [1,2,3]. This leads to myelin loss, axonal loss with variable degrees of axonal pathology, and progressive neurological dysfunction with around 2.5 million affected individuals worldwide [1]. The hallmarks of MS are the formation of lesions (“plaques”) in many areas in the brain and spinal cord [2,3]. Severity and diversity of clinical symptoms of MS largely depend on the frequency and distribution of lesions in the brain and spinal cord. It has a wide range of symptoms including mental, physical, and psychological problems according to the recent Atlas of MS (2013) published by the MS International Federation. The global median prevalence of MS has increased from 30 to 33 cases per 100,000 people from 2008 to 2013 respectively [2,4]. Significantly, the prevalence rate of MS in Jordan increased to 39 per 100,000 people in the period from 2004 to 2005 [5,6,7,8].

The precise cause of MS and the origin of inflammation cascades have not been identified; there are several complicating factors that increase the risk of MS development such as genetic and environmental factors like Epstein–Barr virus (EBV), UV radiation, smoking, and vitamin D deficiency [9,10]. The international Genome Wide Association Scan (GWAS) studies have identified more than 200 independent MS-associated variants, each contributing a small effect to disease risk. One of the most non-human leukocyte antigen (non-HLA) loci is found within the cluster of differentiation 40 (*CD40*) gene [11]. Recent studies support the pathogenic role of CD40 in a number of autoimmune diseases including MS [4]. CD40 belongs to the tumor necrosis factor (TNF) receptor superfamily and is expressed mainly on B cells, microglia, macrophages, and other antigen-presenting cells (APCs) [9,11]. CD40 plays an essential role in the development of normal B cell responses and, therefore, immune regulation and homeostasis [4,11]. Another gene of the non-major histocompatibility complex (MHC) MS genes was recognized as the interleukin 7 receptor gene (*IL7R*), which is translated to a functional cell receptor for Interleukin 7 (IL-7) [12,13]. IL7R plays a significant role in the survival, maintenance, proliferation, and homeostasis of T cells, and may also have a main signalling function through the autoimmunity cascade [14]. Various studies have proved the role of rs6897932 as a risk factor for MS in many European, Japanese, and Caucasian populations [15,16]. Finally, it has been suggested that the lymphocytes activation gene 3 (*LAG3*) also plays a major role as an immune inhibitory receptor, which is mainly found on activated T cells (CD4, CD8, Treg), B cells, and natural killer (NK) cells [17]. The *LAG3* gene is located in the short arm of chromosome 12 at a position of 13.31 and encodes a transmembrane protein that is closely related to the CD40 protein in structure with around 20% amino acid identity level [18].

A better understanding of the disease-associated genes is crucial to predicting how the genetic and non-genetic risk factors interact together to affect the disease’s progression [1,3]. This will improve the development of appropriate treatment plans for each individual patient. This study was conducted to investigate and confirm the potential role and relationship of many polymorphisms of *IL7R*, *LAG3*, and *CD40* genes with different clinical data of MS patients in the Jordanian Arab population.

## 2. Materials and Methods

### 2.1. Population and Experimental Design

This study consisted of 218 Jordanian MS patients recruited from the Jordanian Royal Medical Services hospital, Al-Basheer Hospital, King Abdullah University Hospital, and Princess Basma Hospital. The expanded disability status scale (EDSS) was used to score, measure, and assess patient disability levels. This scale ranges from 0 to 10 increasing by 0.5 unit increments that represent higher levels of disability at the upper end. All scores based on this scale were scored by a neurologist after clinical examination. All patients were given a written consent form and the study was ethically authorized by the Jordan University of Science and Technology Institutional Review Board (IRB) with an ethical code number (12/108/2017).

For a patient to be included in our study, they needed an official confirmed diagnosis of MS and to be a Jordanian resident. Jordanian MS comorbidities, which were observed at the time of diagnosis, included thyroid gland diseases, rheumatoid arthritis, migraine, seizure disorder, familial Mediterranean fever, irritable bowel syndrome, and digestive system problems. Studying comorbidities in patients diagnosed with MS could help improve prognosis and personalized disease management. The exclusion criteria for cases included the following characteristics: patients with unconfirmed diagnosis of MS, patients who had one of their first or second degree family participating in our study, individuals from other nationalities, patients who have a phobia of either blood or needles, patients that had not given informed written consent, or patients with limited or absent recorded clinical data.

### 2.2. Vitamin D Analysis

Whole blood samples were collected from MS patients in plain tubes. 25-hydroxyvitamin D (25(OH)D) was used to evaluate the concentration of 25(OH)D3 in the serum. The serum was analyzed in the medical department laboratories using the ab213966 25(OH) vitamin D enzyme-linked immunosorbent assay (ELISA) kit provided by Abcam (Cambridge, U.K.) by following their instructions. Individuals that were considered deficient for vitamin D have less than 30 nmol/L of 25[OH]D in their serum. This is based on the international guideline treatment for vitamin D deficiency published by the American Association of Clinical Endocrinologists [19].

### 2.3. Genotyping and SNPs Selection

The genomic DNA was extracted from collected blood samples according to the standard kit procedure (the Gentra^®^ Puregene^®^ Blood Kit, Qiagen, Germany). Quality and quantity of the extracted genome were measured using a Nano-Drop ND-1000 (Bio Drop, U.K.) and gel electrophoresis. DNA samples were sent to the Australian Genome Research Facility (AGRF; Melbourne Node, Melbourne, Australia) for genotyping using the Sequenom MassARRAY^®^ system (iPLEX GOLD) (Sequenom, San Diego, CA, USA). The investigated SNPs in this study were: rs6897932, rs13188960, rs1494554, rs987107, rs987106, rs3194051, rs1494571, rs11567705, rs6871748, rs969128, and rs1494555 within *IL7R* gene; rs2365095, rs1922452, rs951818, rs870849, rs188255, rs11227 within the *LAG3* gene; and rs6074022, rs1883832, and rs11086996 within the *CD40* gene.

### 2.4. Statistical Analysis

We used the Statistical Package for Social Sciences (SPSS) version 25.0 (SPSS, Inc., Chicago, IL, USA) to perform Pearson’s χ ^2^-test genotype-phenotype analysis. The odds ratio (OR) with 95% confidence interval (CI) was also calculated to evaluate the risk associated with genotypes/alleles. The multinomial logistic regression was used to predict possible associations between the variables.

## 3. Results

### 3.1. Clinical and Demographic Characteristics of MS Patients

The description of the clinical features of patients is summarized in Table 1. In brief, a total of 67% of the 218 Jordanian MS patients were women and 33% men. The median age of MS patients was 35.5 years with a range of 15 to 64 years. The average age of MS patients at onset was 28.909 ± 8.354 years. The average EDSS score for MS patients at the time of diagnosis was 2.403 ± 1.649. A total of 31 (16%) patients had a family history of MS and 74 (34%) had previously smoked. The screened individuals had four MS forms: 1 (0.5%) patient with radiological isolated syndrome (RIS), 2 (1%) patients with clinically isolated syndrome (CIS), 190 (87%) patients with relapsing-remitting MS (RRMS), and 25 (11.5%) patients with secondary progressive MS (SPMS). Additionally, the screened MS patients were administered disease modifying therapies (DMTs), which included avnoex (9%), rebif (32%), betaferon (33%), fingolimod (23%), and natalizumab (3%). A total of 125 (57%) patients had relapsed once before initial treatment and 71 patients (33%) had more than one relapse. Finally, the majority of MS patients (*n* = 172, 79%) had a low level of vitamin D.

### 3.2. Genetic Association of MS Phenotypes with SNPs of IL7R, LAG3, and CD40 Genes

The association of the investigated IL7R SNPs with several clinical characteristics of MS in Jordanian patients are summarized in Table 2. Three SNPs, rs3194051, rs987107, and rs1494571, were significantly associated with vitamin D deficiency with *P*-values of 0.03, 0.047, and 0.036, respectively. Also, rs3194051 showed a strong association with co-morbidity (*P*-value = 0.003). The rs1494555 SNP had strong correlation with both EDSS at presentation (*p*-value = 0.026) and number of relapses before drug onset (*P*-value = 0.027). rs969128 SNP was the last variant that had a significant association with the number of relapses before drug onset (*P*-value = 0.04). None of the investigated LAG3 SNPs showed any significant association with the clinical features of MS as shown in Table 3, except for rs1922452 polymorphism associated with co-morbidity (*P*-value = 0.028). The association between the investigated CD40 SNPs and the studied MS clinical features are shown in Table 4. The two polymorphisms that were significantly associated with vitamin D deficiency of MS patients were rs6074022 and rs1883832 (*P*-value = 0.049 for both).

## 4. Discussion

MS is a global public health issue posing socio-economic and life quality challenges [5,20]. Although MS is an idiopathic disease, the epidemiology study refers to environmental and genetic factors underlying susceptibility to MS [21]. MS is usually a T-cell-mediated disease where activated T cells play a key role in the pathogenesis of this disease [22,23]. The interaction of these genes (*IL7R*, *CD40* and *LAG3*) with MHC class molecules is associated with MS and other autoimmune diseases [13,24]. Further genetic studies should be conducted to reveal in-depth information about MS genetics because there are insufficient data points in this field of study. In addition, many showed insignificant MS association with the genes variants. Several studies indicated that genetic variations impact not only the susceptibility of MS but also the disease’s clinical course and severity [25,26]. Due to the lack of research studies concerning the association of genetic variants with Jordanian MS patient’s clinical data, a total number of 21 SNPs were studied within three genes (rs6897932, rs13188960, rs1494554, rs987107, rs987106, rs3194051, rs1494571, rs11567705, rs6871748, rs969128, and rs1494555 within *IL7R*; rs2365095, rs1922452, rs951818, rs870849, rs188255, and rs11227 within *LAG3*; and rs6074022, rs1883832, and rs11086996 within *CD40*) in 218 MS patients of the Jordanian Arab population.

Mutations in immune system genes interleukin 7 receptor (*IL7R*), lymphocyte activation 3 gene (*LAG3*), and CD40 co-stimulatory molecule (*CD40*) affect the underlying roles of these genes as immune homeostasis regulatory genes [27,28]. *IL7R* is considered to be one of the key candidate genes for MS because it plays a main role in the regulation of the T cell effector function and the development of functional mature lymphocytes [16,29]. In our study, the association of IL7R SNPs with MS phenotypes revealed five variants that were statistically significant with certain phenotypes of the disease. rs1494555 was significantly associated with patients presenting disability scored by EDSS score (*P*-value = 0.026) and with the number of relapses (*P*-value = 0.027). The rs3194051 variant showed strong association with MS patients who have other chronic medical conditions (comorbidity MS) with *P*-value less than 0.05 (*P*-value = 0.003). Additionally, three variants were associated with vitamin D deficiency (rs987107, rs3194051, and rs1494571). Despite many studies reporting the significant association of rs6897932 with increased MS risk [10], we did not find any significant association with any of the clinical features of MS.

Additionally, we found one variant of the *LAG3* gene (rs1922452, *P*-value = 0.028) associated with MS comorbidity, in contrast with other studies of MS on the Swedish population that revealed no significant correlation between *LAG3* and MS susceptibility [24]. Thus, it was suggested that studying the comorbidity diseases associated with MS may give insights into the pathophysiology, origins, and potential treatment strategies of MS as there have been conflicting results between different populations; however, this can only be confirmed with further studies [30].

In this study, MS patients who had vitamin D deficiency at disease onset exhibited strong association with three SNPs of *IL7R*; rs987107 (*P*-value=0.047), rs3194051 (*P*-value=0.03,) and rs1494571 (*P*-value=0.036), as well as two polymorphisms within the *CD40* gene: rs6074022 and rs1883832 (with a *P*-value=0.049 for both). Many studies reported that MS patients with a vitamin D deficiency both have an increased risk of developing MS and have poorer responses to treatment courses of the disease [31,32]. Hence, it can be inferred that vitamin D is one of the key factors that affect the development of MS [33].

In conclusion, the role of MS candidate genes, including *IL7R*, *LAG3*, and *CD40*, do not only influence disease susceptibility but also have an impact on clinical features. Moreover, highlighting the impact of genetic polymorphisms within candidate genes on the pathways and phenotypes of MS will aid with an earlier diagnosis and better treatment procedures. This will help medical professionals to build knowledge about the factors involved in MS development in different ethnic backgrounds. One of the limitations of this study is that the current research design was based on the use of SNPs in certain genes previously associated with MS in other published cohorts. Nevertheless, it is vital, prior to evaluating the correlation of these SNPs with MS phenotypes, to carry out a case control study first. As not many pharmacogenetic and genetic association studies have been conducted in Jordan [34,35], future research with a larger sample size of both cases and controls to reveal the association of these genes and their polymorphisms with MS risk and prognosis is strongly recommended. Lastly, to define variants involved in the prognosis of patients with MS may also help in stratifying individualized treatment plans.

## Figures and Tables

**Table 1:** Description of clinical characteristics of Jordanian unrelated multiple sclerosis (MS) patients.

Category		Subcategory	Count (n, %)
**Clinical Data**	Sex	Men	72, 33%
		Women	146, 67%
	Age (years) (Mean ± SD *)		35.703 ± 10.199
	Age at MS Onset (years) (Mean ± SD)		28.909 ± 8.354
	EDSS at presentation		2.403 ± 1.649
	Comorbidity	Yes	35, 16%
		No	183, 84%
	Family History of MS	Yes	31, 14%
		No	187, 86%
	Smoking Status	Yes	74, 34%
		No	143, 65%
		Past Smoker	1, 0.5%
	Form of MS	RIS	1, 0.5%
		CIS	2, 1.0%
		RRMS	190, 87%
		SPMS	25, 11.5%
	Type of DMTs	Interferon’s	0, 0%
		Avonex	19, 9%
		Rebif	70, 32%
		Betaferon	72, 33%
		Fingolimod	50, 23%
		Natalizumab	7, 3%
	Number of relapses before medication onset	= 1	125, 57%
		>1	71, 33%
		Unknown	22, 10%
	Vitamin D Deficiency	Yes **	172, 79%
		No	13, 6%
		Not detected	33, 15%

* SD: Standard deviation. ** patients with vitamin D concentration <30 ng/mL were consider to have vitamin D deficiency. RIS: Radiological isolated syndrome, CIS: Clinically isolated syndrome, RRMS: Relapsing-remitting MS, SPMS: Secondary progressive MS.

**Table 2:** Association between single nucleotide polymorphism genotypes of the interleukin 7 receptor gene and the clinical characteristics of multiple sclerosis.

Clinical Characteristics	*IL7R* Gene										
	rs6897932 CC/CT/TT	rs13188960 GG/GT/TT	rs1494554 GG/TG/TT	rs987107 AA/GA/GG	rs987106 AA/AT/TT	rs3194051 AA/AG/GG	rs1494571 CC/GC/GG	rs11567705 CC/CG/GG	rs6871748 CC/TC/TT	rs969128 AA/AG/GG	rs1494555 AA/AG/GG
Body Mass Index **	0.947 ^a^ 0.108 ^b^	0.967 ^a^ 0.068 ^b^	0.495 ^a^ 2.668 ^b^	0.269 ^a^ 2.648 ^b^	0.210 ^a^ 3.146 ^b^	0.225 ^a^ 3.002 ^b^	0.203 ^a^ 3.210 ^b^	0.957 ^a^ 0.088 ^b^	0.112 ^a^ 4.422 ^b^	0.760 ^a^ 0.550 ^b^	0.145 ^a^ 3.904 ^b^
MS Duration **	0.460 ^a^ 1.560 ^b^	0.523 ^a^ 1.300 ^b^	0.241 ^a^ 3.606 ^b^	0.080 ^a^ 5.116 ^b^	0.676 ^a^ 0.784 ^b^	0.120 ^a^ 4.280 ^b^	0.162 ^a^ 3.670 ^b^	0.976 ^a^ 0.048 ^b^	0.470 ^a^ 1.514 ^b^	0.560 ^a^ 1.164 ^b^	0.664 ^a^ 0.820 ^b^
Age at MS Onset **	0.745 ^a^ 0.590 ^b^	0.629 ^a^ 0.930 ^b^	0.448 ^a^ 1.612 ^b^	0.435 ^a^ 1.670 ^b^	0.548 ^a^ 1.206 ^b^	0.673 ^a^ 0.792 ^b^	0.655 ^a^ 0.848 ^b^	0.793 ^a^ 0.464 ^b^	0.870 ^a^ 0.027 ^b^	0.331 ^a^ 2.224 ^b^	0.903 ^a^ 0.204 ^b^
EDSS Score at Presentation **	0.275 ^a^ 2.602 ^b^	0.163 ^a^ 3.666 ^b^	0.464 ^a^ 1.540 ^b^	0.401 ^a^ 1.836 ^b^	0.179 ^a^ 3.472 ^b^	0.504 ^a^ 1.374 ^b^	0.446 ^a^ 1.622 ^b^	0.285 ^a^ 2.526 ^b^	0.177 ^a^ 3.492 ^b^	0.866 ^a^ 0.288 ^b^	0.026 ^a^ 7.420 ^b^
No of Relapses before Drug Treatment *	0.572 ^a^ 1.246 ^b^	0.712 ^a^ 0.920 ^b^	0.530 ^a^ 1.336 ^b^	0.438 ^a^ 1.678 ^b^	0.810 ^a^ 0.433 ^b^	0.634 ^a^ 0.936 ^b^	0.540 ^a^ 1.202 ^b^	0.683 ^a^ 0.766 ^b^	0.933 ^a^ 0.151 ^b^	0.040 ^a^ 6.179 ^b^	0.027 ^a^ 7.167 ^b^
Vit. D Deficiency *	0.416 ^a^ 1.570 ^b^	0.416 ^a^ 1.570 ^b^	0.080 ^a^ 4.665 ^b^	0.047 ^a^ 6.149 ^b^	0.114 ^a^ 4.288 ^b^	0.030 ^a^ 7.129 ^b^	0.036 ^a^ 6.415 ^b^	0.001 ^a^ 2.378 ^b^	0.262 ^a^ 3.100 ^b^	0.887 ^a^ 0.239 ^b^	0.124 ^a^ 4.104 ^b^
Patient Age **	0.988 ^a^ 0.024 ^b^	0.931 ^a^ 0.144 ^b^	0.790 ^a^ 2.076 ^b^	0.500 ^a^ 1.390 ^b^	0.489 ^a^ 1.434 ^b^	0.316 ^a^ 2.318 ^b^	0.560 ^a^ 1.164 ^b^	0.925 ^a^ 0.156 ^b^	0.732 ^a^ 0.626 ^b^	0.305 ^a^ 2.386 ^b^	0.918 ^a^ 0.170 ^b^
Family History *	0.327 ^a^ 2.066 ^b^	0.328 ^a^ 1.921 ^b^	0.170 ^a^ 3.566 ^b^	0.310 ^a^ 2.362 ^b^	0.181 ^a^ 3.614 ^b^	0.193 ^a^ 3.477 ^b^	0.212 ^a^ 3.181 ^b^	0.379 ^a^ 2.061 ^b^	0.336 ^a^ 2.096 ^b^	0.749 ^a^ 0.743 ^b^	0.066 ^a^ 5.466 ^b^
Comorbidity *	0.356 ^a^ 2.052 ^b^	0.400 ^a^ 1.876 ^b^	0.688 ^a^ 0.726 ^b^	0.309 ^a^ 2.495 ^b^	0.747 ^a^ 0.605 ^b^	0.003 ^a^ 2.208 ^b^	0.580 ^a^ 1.021 ^b^	0.926 ^a^ 0.282 ^b^	0.338 ^a^ 1.820 ^b^	0.565 ^a^ 1.222 ^b^	0.676 ^a^ 0.812 ^b^
Smoking *	0.959 ^a^ 0.083 ^b^	0.957 ^a^ 0.088 ^b^	0.306 ^a^ 4.376 ^b^	0.250 ^a^ 2.813 ^b^	0.438 ^a^ 1.688 ^b^	0.297 ^a^ 2.492 ^b^	0.324 ^a^ 4.227 ^b^	0.907 ^a^ 0.274 ^b^	0.985 ^a^ 0.031 ^b^	0.886 ^a^ 0.452 ^b^	0.743 ^a^ 0.596 ^b^
Form of MS *	0.948 ^a^ 1.973 ^b^	0.819 ^a^ 2.929 ^b^	0.860 ^a^ 5.620 ^b^	0.869 ^a^ 4.148 ^b^	0.811 ^a^ 5.082 ^b^	0.915 ^a^ 3.413 ^b^	0.815 ^a^ 6.124 ^b^	0.626 ^a^ 1.710 ^b^	0.668 ^a^ 1.904 ^b^	0.698 ^a^ 1.830 ^b^	0.422 ^a^ 3.961 ^b^
Sex *	0.703 ^a^ 0.888 ^b^	0.787 ^a^ 0.521 ^b^	0.785 ^a^ 0.503 ^b^	0.955 ^a^ 0.147 ^b^	0.582 ^a^ 1.049 ^b^	0.912 ^a^ 0.243 ^b^	0.896 ^a^ 0.250 ^b^	0.580 ^a^ 1.093 ^b^	0.190 ^a^ 3.565 ^b^	0.539 ^a^ 1.418 ^b^	0.939 ^a^ 0.113 ^b^

a: *p*-Value < 0.05 is considered significant, b: Chi-squared value. * Pearson’s chi-squared test was used to determine genotype-phenotype association. ** Analysis of variance (ANOVA) test was used to determine genotype-phenotype association.

**Table 3:** Association between single nucleotide polymorphism genotypes of the lymphocytes activation gene 3 and the clinical characteristics of multiple sclerosis.

Clinical Characteristics	*LAG3* Gene					
	rs2365095 CC/CT/TT	rs1922452 AA/GA/GG	rs951818 AA/AC/CC	rs870849 CC/TC/TT	rs188255 CC/GC/GG	rs11227 GG/TG/TT
Body Mass Index **	0.281 ^a^ 2.554 ^b^	0.358 ^a^ 2.068 ^b^	0.320 ^a^ 2.290 ^b^	0.397 ^a^ 1.856 ^b^	0.340 ^a^ 2.168 ^b^	0.699 ^a^ 0.718 ^b^
MS Duration **	0.811 ^a^ 0.420 ^b^	0.942 ^a^ 0.118 ^b^	0.798 ^a^ 0.452 ^b^	0.716 ^a^ 0.668 ^b^	0.880 ^a^ 0.256 ^b^	0.647 ^a^ 0.872 ^b^
Age at MS Onset **	0.842 ^a^ 0.346 ^b^	0.861 ^a^ 0.300 ^b^	0.598 ^a^ 1.030 ^b^	0.555 ^a^ 1.180 ^b^	0.973 ^a^ 0.054 ^b^	0.834 ^a^ 0.364 ^b^
EDSS Score at Presentation **	0.367 ^a^ 2.012 ^b^	0.795 ^a^ 0.460 ^b^	0.791 ^a^ 0.470 ^b^	0.954 ^a^ 0.094 ^b^	0.509 ^a^ 1.354 ^b^	0.280 ^a^ 2.564 ^b^
No. of Relapses before Drug Treatment *	0.725 ^a^ 0.812 ^b^	0.112 ^a^ 4.433 ^b^	0.294 ^a^ 2.485 ^b^	0.980 ^a^ 0.078 ^b^	0.483 ^a^ 1.810 ^b^	0.313 ^a^ 2.262 ^b^
Vit. D Deficiency *	0.253 ^a^ 2.658 ^b^	0.664 ^a^ 0.888 ^b^	0.789 ^a^ 0.629 ^b^	0.444 ^a^ 1.926 ^b^	0.332 ^a^ 2.292 ^b^	0.953 ^a^ 0.096 ^b^
Patient Age **	0.973 ^a^ 0.056 ^b^	0.582 ^a^ 1.084 ^b^	0.248 ^a^ 2.812 ^b^	0.359 ^a^ 2.060 ^b^	0.669 ^a^ 0.806 ^b^	0.803 ^a^ 0.438 ^b^
Family History *	0.833 ^a^ 0.471 ^b^	0.291 ^a^ 2.555 ^b^	0.485 ^a^ 1.460 ^b^	0.353 ^a^ 2.120 ^b^	0.438 ^a^ 1.817 ^b^	0.285 ^a^ 2.613 ^b^
Comorbidity *	0.799 ^a^ 0.436 ^b^	0.028 ^a^ 6.923 ^b^	0.089 ^a^ 4.869 ^b^	0.130 ^a^ 4.071 ^b^	0.481 ^a^ 1.801 ^b^	0.994 ^a^ 0.012 ^b^
Smoking *	0.656 ^a^ 1.057 ^b^	0.476 ^a^ 1.548 ^b^	0.727 ^a^ 0.666 ^b^	0.561 ^a^ 1.138 ^b^	0.925 ^a^ 0.283 ^b^	0.945 ^a^ 0.140 ^b^
Form of MS *	0.620 ^a^ 2.342 ^b^	0.467 ^a^ 3.724 ^b^	0.503 ^a^ 3.567 ^b^	0.305 ^a^ 6.873 ^b^	0.070 ^a^ 6.924 ^b^	0.577 ^a^ 4.104 ^b^
Sex *	0.539 ^a^ 1.316 ^b^	0.879 ^a^ 0.275 ^b^	0.939 ^a^ 0.139 ^b^	0.727 ^a^ 0.711 ^b^	0.548 ^a^ 1.218 ^b^	0.551 ^a^ 1.188 ^b^

a: *P*-Value < 0.05 is considered significant, b: Chi-squared value. * Pearson’s Chi-squared test was used to determine genotype-phenotype association. ** Analysis of variance (ANOVA) test was used to determine genotype-phenotype association.

**Table 4:** Association between single nucleotide polymorphism genotypes of the cluster of differentiation 40 gene and the clinical characteristics of multiple sclerosis.

Clinical Characteristics	*CD40* Gene		
	rs6074022 CC/TC/TT	rs1883832 CC/CT/TT	rs11086996 CC/CT/TT
Body Mass Index **	0.894 ^a^ 0.226 ^b^	0.894 ^a^ 0.226 ^b^	0.120 ^a^ 4.278 ^b^
MS Duration **	0.588 ^a^ 1.066 ^b^	0.607 ^a^ 1.000 ^b^	0.230 ^a^ 2.964 ^b^
Age at MS Onset **	0.261 ^a^ 2.702 ^b^	0.230 ^a^ 2.964 ^b^	0.161 ^a^ 3.692 ^b^
EDSS Score at Presentation **	0.870 ^a^ 0.278 ^b^	0.870 ^a^ 0.278 ^b^	0.540 ^a^ 1.236 ^b^
No. of Relapses before Drug Treatment *	0.702 ^a^ 0.689 ^b^	0.702 ^a^ 0.689 ^b^	0.150 ^a^ 3.752 ^b^
Vit. D Deficiency *	0.049 ^a^ 6.342 ^b^	0.049 ^a^ 6.342 ^b^	0.750 ^a^ 0.576 ^b^
Patient Age **	0.345 ^a^ 2.140 ^b^	0.345 ^a^ 2.140 ^b^	0.453 ^a^ 1.592 ^b^
Family History *	0.571 ^a^ 1.151 ^b^	0.571 ^a^ 1.151 ^b^	0.706 ^a^ 0.669 ^b^
Comorbidity *	0.556 ^a^ 1.375 ^b^	0.556 ^a^ 1.375 ^b^	0.265 ^a^ 2.530 ^b^
Smoking *	0.994 ^a^ 0.013 ^b^	0.994 ^a^ 0.013 ^b^	0.771 ^a^ 0.594 ^b^
Form of MS *	0.895 ^a^ 2.278 ^b^	0.868 ^a^ 0.990 ^b^	0.289 ^a^ 6.198 ^b^
Sex *	0.468 ^a^ 1.535 ^b^	0.468 ^a^ 1.535 ^b^	0.497 ^a^ 1.565 ^b^

a: *P*-Value < 0.05 is considered significant, b: Chi-squared value. * Pearson’s chi-squared test was used to determine genotype-phenotype association. ** Analysis of variance (ANOVA) test was used to determine genotype-phenotype association.

## References

[1] Franklin G.M., Nelson L. (2003). Environmental risk factors in multiple sclerosis: Causes, triggers, and patient autonomy. Neurology.

[2] Palmer A.M. (2013). Multiple sclerosis and the blood-central nervous system barrier. Cardiovasc. Psychiatry Neurol..

[3] Baranzini S.E., Oksenberg J.R. (2017). The Genetics of Multiple Sclerosis: From 0 to 200 in 50 Years. Trends Genet..

[4] Chen D., Ireland S.J., Remington G., Alvarez E., Racke M.K., Greenberg B., Frohman E.M., Monson N.L. (2016). CD40-mediated NF-κB activation in B cells is increased in multiple sclerosis and modulated by therapeutics. J. Immunol..

[5] Kurtzke J.F. (2015). On the epidemiology of multiple sclerosis in the iddle East and North Africa. Neuroepidemiology.

[6] Adoni T. (2016). Immunogenetics of multiple sclerosis: More questions than answers. Arquivos de neuro-psiquiatria.

[7] Ascherio A., Munger K.L. (2007). Environmental risk factors for multiple sclerosis. Part II: Noninfectious factors. Ann. Neurol..

[8] Compston A., Coles A. (2008). Multiple sclerosis. Lancet (London, England).

[9] Sokolova E.A., Malkova N.A., Korobko D.S., Rozhdestvenskii A.S., Kakulya A.V., Khanokh E.V., Delov R.A., Platonov F.A., Popova T.Y., Aref E.G. (2013). Association of SNPs of CD40 gene with multiple sclerosis in Russians. PLoS ONE.

[10] Liu H., Huang J., Dou M., Liu Y., Xiao B., Liu X., Huang Z. (2017). Variants in the IL7RA gene confer susceptibility to multiple sclerosis in Caucasians: Evidence based on 9734 cases and 10436 controls. Sci. Rep..

[11] Blanco-Kelly F., Matesanz F., Alcina A., Teruel M., Díaz-Gallo L.M., Gómez-García M., López-Nevot M.A., Rodrigo L., Nieto A., Cardeña C. (2010). CD40: Novel association with Crohn’s disease and replication in multiple sclerosis susceptibility. PLoS ONE..

[12] Zuvich R.L., McCauley J.L., Oksenberg J.R., Sawcer S.J., De Jager P.L., Aubin C., Cross A.H., Piccio L., Aggarwal N.T., International Multiple Sclerosis Genetics Consortium (2010). Genetic variation in the IL7RA/IL7 pathway increases multiple sclerosis susceptibility. Hum. Genet..

[13] Galarza-Muñoz G., Briggs F., Evsyukova I., Schott-Lerner G., Kennedy E.M., Nyanhete T., Wang L., Bergamaschi L., Widen S.G., Tomaras G.D. (2017). Human Epistatic Interaction Controls IL7R Splicing and Increases Multiple Sclerosis Risk. Cell.

[14] Dooms H. (2013). Interleukin-7: Fuel for the autoimmune attack. J. Autoimmun..

[15] Sayad A., Solgi G., Noroozi R., Arsang-Jang S., Inoko H., Taheri M. (2017). Interleukin 7 receptor alpha gene variants are correlated with gene expression in patients with relapsing-remitting multiple sclerosis. Iran J. Allergy Asthma Immunol..

[16] Traboulsee A.L., Bernales C.Q., Ross J.P., Lee J.D., Sadovnick A.D., Vilariño-Güell C. (2014). Genetic variants in IL2RA and IL7R affect multiple sclerosis disease risk and progression. Neurogenetics.

[17] Triebel F., Jitsukawa S., Baixeras E., Roman-Roman S., Genevee C., Viegas-Pequignot E., Hercend T. (1990). LAG-3, a novel lymphocyte activation gene closely related to CD4. J. Exp. Med..

[18] Wang J., Sanmamed M.F., Datar I., Su T.T., Ji L., Sun J., Chen L., Chen Y., Zhu G., Yin W. (2019). Fibrinogen-like Protein 1 Is a Major Immune Inhibitory Ligand of LAG-3. Cell.

[19] Camacho P.M., Petak S.M., Binkley N., Clarke B.L., Harris S.T., Hurley D.L., Kleerekoper M., Lewiecki E.M., Miller P.D., Narula H.S. (2016). American Association of Clinical Endocrinologists and American College of Endocrinology Clinical Practice Guidelines for the Diagnosis and Treatment of Postmenopausal Osteoporosis - 2016. Endocr. Pract..

[20] Giunti G., Fernández E.G., Zubiete E.D., Romero O.R. (2018). Supply and demand in mHealth apps for persons with multiple sclerosis: Systematic search in app stores and scoping literature review. JMIR mHealth uHealth..

[21] Yamabe K., DiBonaventura M.D., Pashos C.L. (2019). Health-related outcomes, health care resource utilization, and costs of multiple sclerosis in Japan compared with US and five EU countries. Clinicoecon. Outcomes Res..

[22] Pettinelli C.B., McFarlin D.E. (1981). Adoptive transfer of experimental allergic encephalomyelitis in SJL/J mice after in vitro activation of lymph node cells by myelin basic protein: Requirement for Lyt 1+ 2- T lymphocytes. J. Immunol..

[23] Heydarpour P., Khoshkish S., Abtahi S., Moradi-Lakeh M., Sahraian M.A. (2015). Multiple sclerosis epidemiology in Middle East and North Africa: A systematic review and meta-analysis. Neuroepidemiology.

[24] Andrews L.P., Marciscano A.E., Drake C.G., Vignali D.A.A. (2017). LAG3 (CD223) as a cancer immunotherapy target. Immunol. Rev..

[25] Shahbazi M., Ebadi H., Fathi D., Roshandel D., Mahamadhoseeni M., Rashidbaghan A., Mahammadi N., Mahammadi M.R., Zamani M. (2009). CCR5-Delta32 allele is associated with the risk of developing multiple sclerosis in the Iranian population. Cell Mol. Neurobiol..

[26] Hong Y., Tang H.R., Ma M., Chen N., Xie X., He L. (2019). Multiple sclerosis and stroke: A systematic review and meta-analysis. BMC Neurol..

[27] Zhang S.M., Willett W.C., Hernán M.A., Olek M.J., Ascherio A. (2000). Dietary Fat in Relation to Risk of Multiple Sclerosis among Two Large Cohorts of Women. Am. J. Epidemiol..

[28] Zaiss M.M., Axmann R., Zwerina J., Polzer K., Gückel E., Skapenko A., Schulze-Koops H., Horwood N., Cope A., Schett G. (2007). Treg cells suppress osteoclast formation: A new link between the immune system and bone. Arthritis Rheum..

[29] Jiang Q., Li W.Q., Aiello F.B., Mazzucchelli R., Asefa B., Khaled A.R., Durum S.K. (2005). Cell biology of IL-7, a key lymphotrophin. Cytokine Growth Factor Rev..

[30] Marrie R.A., Horwitz R.I. (2010). Emerging effects of comorbidities on multiple sclerosis. Lancet Neurol..

[31] Sintzel M.B., Rametta M., Reder A.T. (2018). Vitamin D and multiple sclerosis: A comprehensive review. Neurol. Ther..

[32] Jagannath V.A., Fedorowicz Z., Asokan G.V., Robak E.W., Whamond L. (2018). Vitamin D for the management of multiple sclerosis. Cochrane. Database Syst. Rev..

[33] Speer G. (2013). Impact of vitamin D in neurological diseases and neurorehabilitation: From dementia to multiple sclerosis. Part I: The role of vitamin D in the prevention and treatment of multiple sclerosis. Ideggyogy Sz..

[34] AL-Eitan L., Haddad Y. (2014). Emergence of pharmacogenomics in academic medicine and public health in Jordan: History, present state and prospects. Curr. Pharmacogenom. Pers. Med..

[35] AL-Eitan L., Tarkhan A. (2014). Practical challenges and translational issues in pharmacogenomics and personalized medicine from 2010 onwards. Curr. Pharmacogenom. Pers. Med..

